# Bioengineered model of the human motor unit with physiologically functional neuromuscular junctions

**DOI:** 10.1038/s41598-021-91203-5

**Published:** 2021-06-03

**Authors:** Rowan P. Rimington, Jacob W. Fleming, Andrew J. Capel, Patrick C. Wheeler, Mark P. Lewis

**Affiliations:** National Centre for Sport and Exercise Medicine, School of Sport, Exercise and Health Sciences, Loughborough University, Loughborough, LE11 3TU Leicestershire UK

**Keywords:** Tissue engineering, Musculoskeletal models, Neurological models

## Abstract

Investigations of the human neuromuscular junction (NMJ) have predominately utilised experimental animals, model organisms, or monolayer cell cultures that fail to represent the physiological complexity of the synapse. Consequently, there remains a paucity of data regarding the development of the human NMJ and a lack of systems that enable investigation of the motor unit. This work addresses this need, providing the methodologies to bioengineer 3D models of the human motor unit. Spheroid culture of iPSC derived motor neuron progenitors augmented the transcription of OLIG2, ISLET1 and SMI32 motor neuron mRNAs ~ 400, ~ 150 and ~ 200-fold respectively compared to monolayer equivalents. Axon projections of adhered spheroids exceeded 1000 μm in monolayer, with transcription of SMI32 and VACHT mRNAs further enhanced by addition to 3D extracellular matrices in a type I collagen concentration dependent manner. Bioengineered skeletal muscles produced functional tetanic and twitch profiles, demonstrated increased acetylcholine receptor (AChR) clustering and transcription of MUSK and LRP4 mRNAs, indicating enhanced organisation of the post-synaptic membrane. The number of motor neuron spheroids, or motor pool, required to functionally innervate 3D muscle tissues was then determined, generating functional human NMJs that evidence pre- and post-synaptic membrane and motor nerve axon co-localisation. Spontaneous firing was significantly elevated in 3D motor units, confirmed to be driven by the motor nerve via antagonistic inhibition of the AChR. Functional analysis outlined decreased time to peak twitch and half relaxation times, indicating enhanced physiology of excitation contraction coupling in innervated motor units. Our findings provide the methods to maximise the maturity of both iPSC motor neurons and primary human skeletal muscle, utilising cell type specific extracellular matrices and developmental timelines to bioengineer the human motor unit for the study of neuromuscular junction physiology.

## Introduction

The neuromuscular junction (NMJ) is the specialised synapse between the post-synaptic skeletal muscle fibre and pre-synaptic terminal of the efferent motor nerve axon. Its function is to elicit contraction of the peripheral musculature and consequently control locomotion of the skeleton via a co-ordinated mechanosensory-motor circuit. The NMJ comprises an organised assembly of protein complexes (Agrin-Lrp4-MuSK-Dok7) that form the highly specialised synaptic basal lamina; the function of which is to stabilise the synapse and facilitate the intricate biochemical processes that initiate muscular excitability and contraction^1,2^. Neuronal action potential induces an influx of calcium ions at the motor nerve terminal that elicits fusion of synaptic vesicles at active zones on the terminal membrane. Vesicle fusion mediates the release of the neurotransmitter acetylcholine (ACh) which diffuses through the synaptic cleft and binds nicotinic acetylcholine receptors (AChR) on the post-synaptic membrane^3^. This depolarisation opens voltage-gated sodium ion channels and generates the action potential required for excitation contraction coupling and muscular contraction^4^.

To date, the study of neuromuscular development and physiological function has predominantly utilised experimental rodent, zebrafish and drosophila models^5–7^. As the NMJ is implicated in a variety of neuromuscular/neurodegenerative diseases, multiple transgenic rodent models have been produced to investigate disease specific pathology^8,9^. Although significant advancements have been made utilising these animal models, issues remain when translating animal data to human physiology^10^. This is likely a consequence of subtle differences in the cellular and molecular composition of the human NMJ compared to model animals. Compact NMJ morphologies, reduced relative size compared to skeletal muscle fibre diameter, distribution of active zone proteins, low quanta release, and increased post-synaptic junctional folds are all features of the human NMJ that differ across species^11–15^. Consequently, there remains a requirement to develop representative models of the human NMJ that enable the temporal analysis of the synapse in development, disease, and in response to external stimuli.

Human cellular systems that enable the dynamic modelling of the NMJ have been significantly advanced by the ability to generate human motor neurons from induced pluripotent stem cells (iPSCs)^16,17^. Several systems and methodologies have now been reported that combine human skeletal muscle and iPSC motor neurons in monolayer^18,19^. Specific advances include the first functional model for NMJ drug dose response, modelling of neuromuscular disease, and development of MEMS devices that enable the separate culture and medium isolation of neuronal and myogenic cell types^20–22^. While this is a significant advantage, two-dimensional (2D) systems lack the ability to truly represent the physiological complexity of the NMJ. Three-dimensional (3D) co-culture systems enable the advanced modelling of the neuromuscular system due to the incorporation of extracellular matrix proteins and mechanical forces that enable enhanced biological development^23^. Further, 3D tissue systems provide a flexibility in manipulation (genetic modification/exogenous treatments), throughput, and rapid experimental iteration that cannot be achieved in animal models. 3D microfluidic systems using C2C12 skeletal myoblasts innervated with iPSC motor neurons^24^, and classical engineered tissues using primary rat derived muscle and motor neurons have been reported^25,26^. However, neither system fully utilises human cellular materials and consequently cannot replicate human NMJ physiology.

Significant progress over the last two years has produced three contrasting systems that enable the functional innervation of 3D human skeletal muscle tissue^27^. Osaki et al. utilised a bespoke microfluidic device enabling the separate maturation of iPSC muscle tissue and motor neuron spheroids derived from healthy and amyotrophic lateral sclerosis (ALS) patients^28^. This system was later used to report the first isogenic NMJ using motor neurons derived from iPSCs of reprogrammed commercial primary myoblasts^29^. Although excellent functional evaluation was performed, limited biochemical characterisation of pre- and post-synaptic makers of NMJ formation were evidenced. iPSC myogenic precursors are ideal for autologous disease modelling, however, neither iPSC or commercial myoblasts contain the endogenous supporting populations evident within biopsied primary human skeletal muscle that contribute to its physiology and function. Afshar Bakooshli et al. produced engineered neuromuscular tissues using biopsied primary human skeletal muscle and embryonic stem cell (ESC) derived motor neurons, demonstrating developmental AChƴ to AChɛ subunit switching and myasthenia gravis (MG) disease modelling^30^. However, motor neuron clusters were harvested from monolayer cell cultures and combined with myogenic precursors at experimental onset, using extracellular matrices specifically tailored for skeletal muscle. Self-organising neuromuscular trunk organoids using neuromesodermal progenitors from ESCs and iPSCs have also been reported, evidencing the first inclusion of supporting neuromuscular cell types such as glia and interneurons^31^. However, cultured in a low adherence multiwell plate, this system lacks the extracellular matrix components and longitudinal tension that is critical for skeletal muscle function. To date, no 3D bioengineered neuromuscular system has been produced that uses biopsied primary myoblasts, has tailored extracellular matrices for both motor neuron (type I collagen) and muscle (type I collagen/basement membrane proteins), enables the separate maturation of motor neuron and muscle cultures in 3D, and allows precision in the time of neuronal addition dependent on the stage of myogenesis. Further, while previous work has focussed on disease modelling (ALS, MG), no human model has evidenced functional changes indicative of enhanced muscular excitability following innervation for the study of physiological neuromuscular contraction.

Here, we present the methodologies to produce 3D bioengineered tissues of the human neuromuscular motor unit with physiologically functional NMJs, using primary skeletal muscle and iPSC derived motor neurons. Producing 3D spheroid cultures of motor neurons accelerates development and enhances the transcription of SMI32 mRNAs, as an indicative marker of motor neuron maturity^17,32,33^, 200-fold compared to monolayer equivalents, extending axons > 1000 μm in length. Optimising type I collagen matrices further enhances motor neuron spheroid maturation sixfold and provides a loading mechanism for delivery to primary human skeletal muscle tissue within a freely available, open-source 3D printed system^34^. The separate maturation of both neuronal and myogenic populations allows for control over the motor neuron addition to engineered muscle depending on stage of developmental myogenesis. Analysis of spontaneously occurring twitch force determines the motor pool required to achieve functional innervation in this system. This process has been confirmed to be driven by the motor nerve via antagonistic blocking of the AChR, and co-localisation of pre-synaptic nerve terminals and post-synaptic AChRs. Further, this research evidences enhanced neuromuscular physiology following innervation, through direct quantification of time to peak contraction and half relaxation times in engineered tissues.

## Results

### 3D spheroid culture of iPSC derived motor neuron progenitors accelerates development and enhances maturation

**Figure 1:**
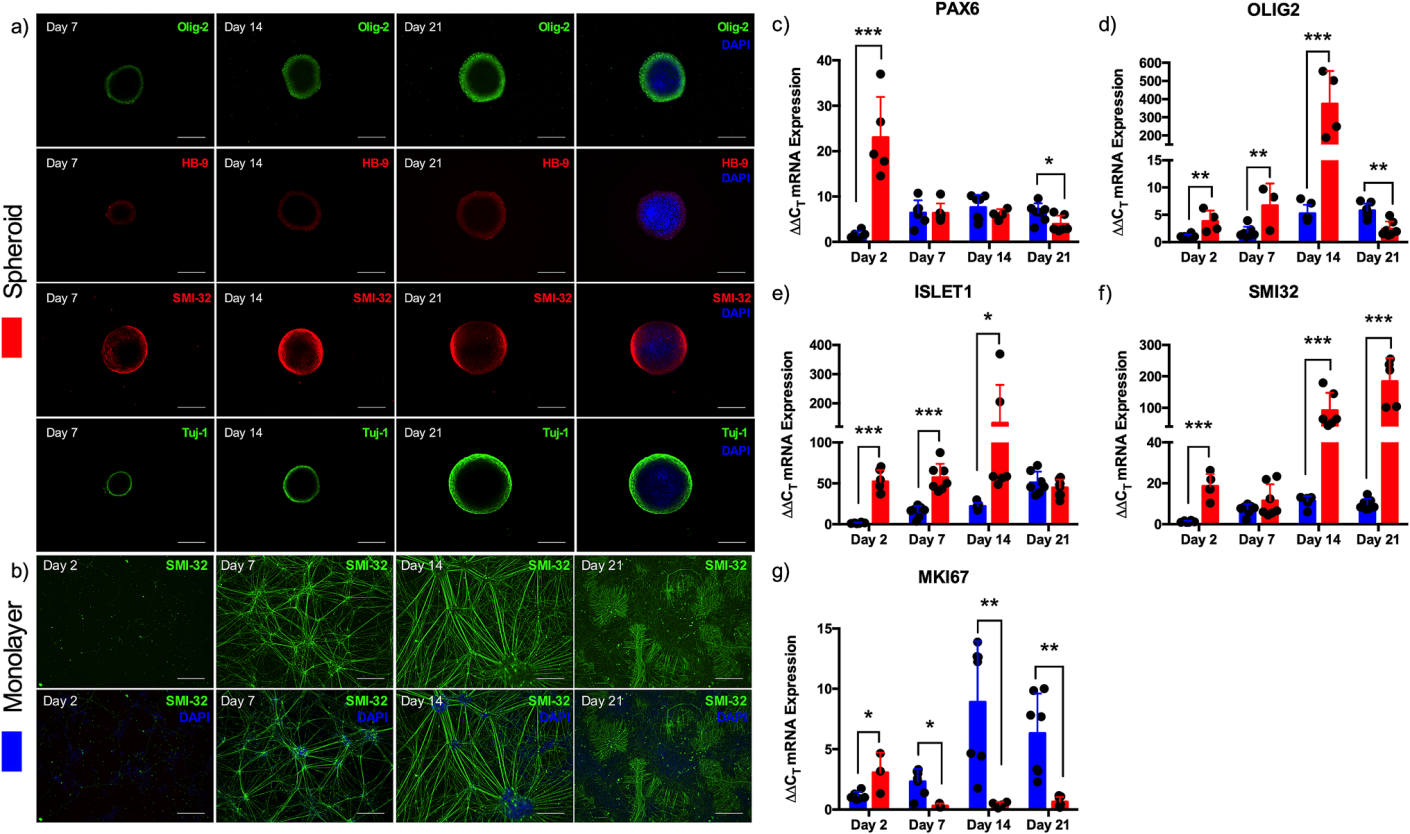
Spheroid culture of iPSC derived motor neuron progenitors accelerates transcription of developmental and mature mRNAs. (**a**) Motor neuron progenitors cultured as 3D spheroids for 7, 14 and 21 days positive for Olig-2, HB-9, SMI-32 and Tuj-1. (**b**) Motor neuron progenitor cells cultured in monolayer for 2, 7, 14 and 21 days positive for SMI-32. (**c**) mRNA gene expression for neuronal stem cell marker PAX6, (**d**) progenitor marker OLIG2, (**e**) motor neuron marker ISLET1, (**f**) mature motor neuron marker SMI32 and (**g**) proliferative marker MKI67. Blue bars = Monolayer (2D), Red bars = Spheroid (3D). Data presented ± standard deviation (SD). *P ≤ 0.05, **P ≤ 0.01, and ***P ≤ 0.001. Scale bars = 200 µm.

To enhance maturity of iPSC motor neurons, progenitor cells were combined within microplates to form developmental spheroids and compared against progenitors cultured in monolayer. iPSC derived motor neuron progenitor spheroids, positive for Olig-2, HB-9, SMI-32 and cytoskeletal marker Tuj-1 enabled continual development across a 3 week culture period (Fig. 1a). In comparison, monolayer cultures also developed positivity of mature marker SMI-32, however, by week 3 adherent cells were observed to be losing phenotype and adherence to coated culture surfaces (Fig. 1b). Transcriptional analysis of motor neuron lineage outlined 20-fold increases in developmental regulator PAX6 mRNA after 2 days spheroid culture (P ≤ 0.001), decreasing thereafter. Contrastingly, monolayer progenitor PAX6 mRNA expression increased from day 2 to day 7 and maintained this elevated level throughout 3 weeks culture, significantly compared to 3D spheroids after 21 days (P ≤ 0.05, Fig. 1c). Expression of progenitor marker OLIG2 mRNA was significantly elevated in spheroid cultures after 2 (P ≤ 0.01) and 7 (P ≤ 0.01) days, peaking at 400-fold after 14 days culture (P ≤ 0.001) prior to decreasing below that of monolayer cells after 21 days (P ≤ 0.01, Fig. 1d). This transcriptional pattern was also evident in motor neuron maker ISLET1 mRNA being significantly elevated in spheroid cultures after 2 (P ≤ 0.001), 7 (P ≤ 0.001) and 14 days (P ≤ 0.05) in culture (Fig. 1e). mRNA of mature motor neuron marker SMI32 was also significantly elevated after 2 days (P ≤ 0.001) in spheroid culture, however continued to increase in transcription throughout development after 14 (P ≤ 0.001) and 21 days (P ≤ 0.001, Fig. 1f), 200-fold compared to monolayer. Proliferative maker MKI67 mRNA was elevated after 2 days in motor neuron spheroids (P ≤ 0.05), however this response was then decreased after 7 (P ≤ 0.05), 14 (P ≤ 0.01) and 21 days culture (P ≤ 0.01), with monolayer populations demonstrating increased proliferation throughout development (Fig. 1g).

### iPSC motor neuron spheroids preferentially extend axons at different stages of development

**Figure 2:**
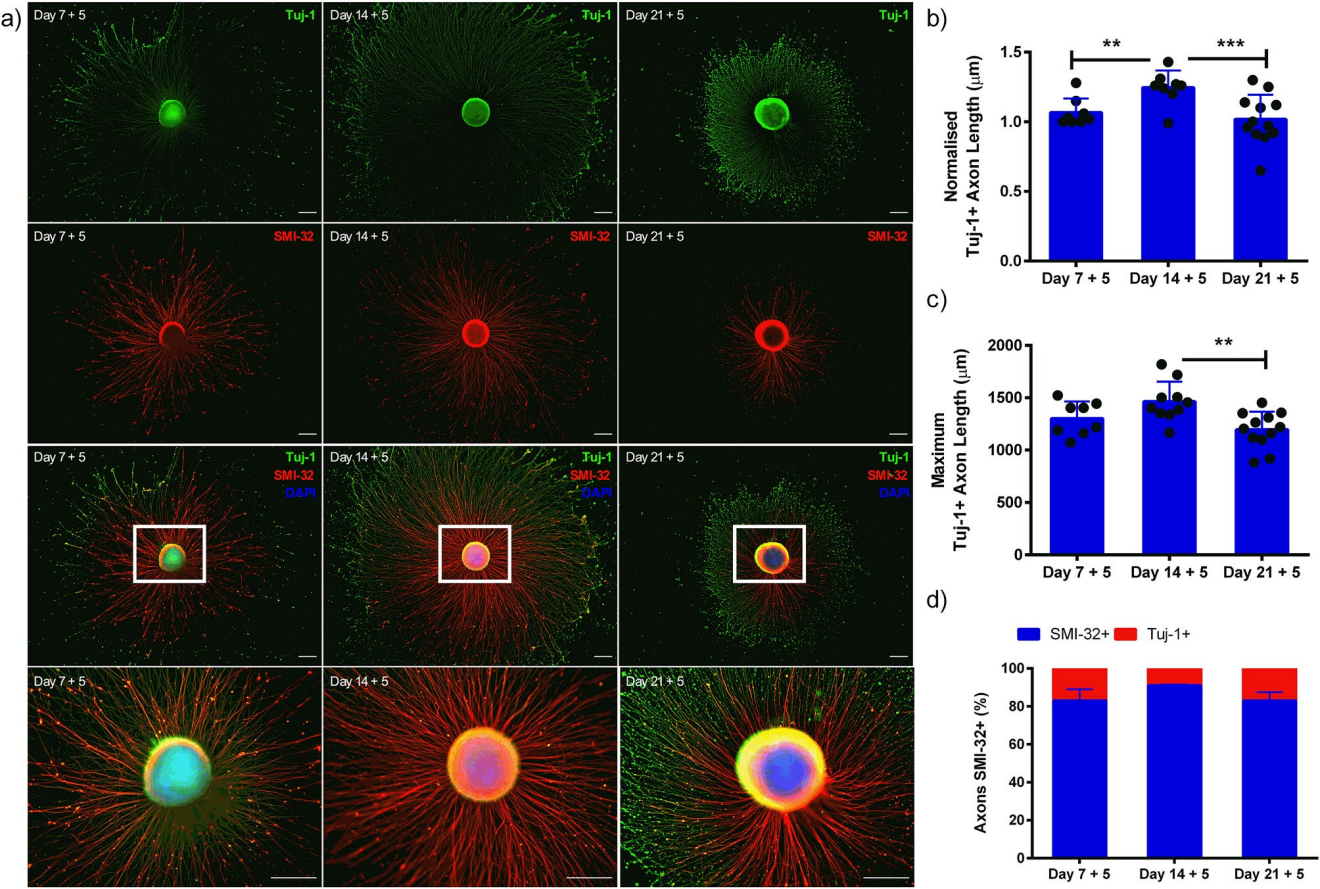
Axon extension of iPSC motor neuron spheroids is influenced by developmental stage. (a) Motor neuron progenitors cultured as 3D spheroids at different stages of development (7, 14 and 21 days) adhered in monolayer culture extend axons over 5 days, e.g. nomenclature ‘7 + 5’ denotes 7 days spheroid culture followed by 5 days adhered monolayer culture. (b) Tuj-1 positive (+) axon length after 5 days adherent culture following seeding at days 7, 14 and 21 days of maturation. Axon length data normalised to day 7 + 5 time-point. (**c**) Maximum Tuj-1 axon lengths (µm) at each stage of maturation. (d) Percentage (%) axons positive for mature heavy neurofilament SMI-32 after 5 days adherent culture at different development stages. Data indicative of minimum n = 8 per time-point and presented ± standard deviation (SD). **P ≤ 0.01, and ***P ≤ 0.001. Scale bars = 200 µm.

To investigate the axonal extension potential of motor neurons at different developmental stages, spheroids were removed after 7, 14, and 21 days of differentiation, adhered to culture well plates and left for 5 days (Fig. 2a). Spheroids extended significantly longer axons after 14 days of development, compared to 7 (P ≤ 0.01) and 21 days (Fig. 2b, P ≤ 0.001) indicating that extension of Tuj-1 + axons is influenced by developmental stage (P ≤ 0.001). Maximum lengths of axons at all three developmental stages typically exceeded 1000 µm, with axons significantly longer after 14 days compared to 21 days (P ≤ 0.01). SMI-32 positivity of spheroid axons remains consistent (> 80%) at all stages of development (P > 0.05 Fig. 2d), indicating comparable high levels of axon maturity. Considering previous mRNA expression with axon extension data, it was determined that 14 days spheroid culture was optimal for motor neuron development before adherent culture. However, both earlier and later time-points produced viable motor neurons of enhanced development extending long axons.

### Culture of iPSC motor neuron spheroids within 3D collagen I matrices enhances expression of mature motor neuron mRNAs

**Figure 3:**
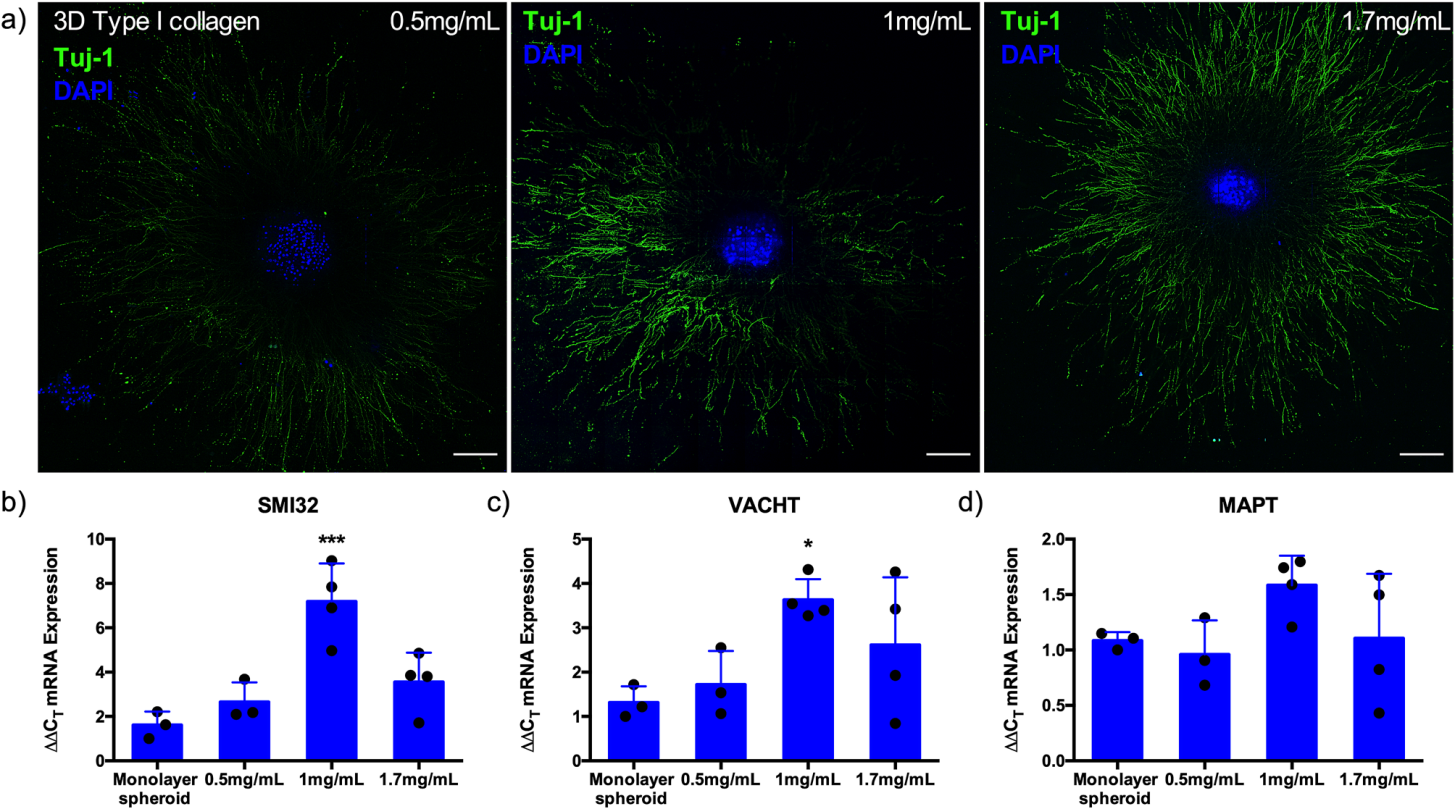
Culture of iPSC motor neuron spheroids within 3D collagen I matrices enhances mRNA expression of heavy neurofilament (SMI-32), vesicular transporter of acetylcholine (VAChT) and axonal extension (MAPT). (**a**) Confocal tile scans of motor neuron progenitor spheroids suspended within 3D collagen I matrices of different concentrations (0.5, 1 and 1.7 mg/mL) adhere and extend axons over 5 days. (**b**) mRNA expression of mature motor neuron markers SMI32 and (**c**) VACHT, and (**d**) MAPT as indication of amount of axonal extension compared to adhered monolayer spheroids. Data presented ± standard deviation (SD). *P ≤ 0.05 and ***P ≤ 0.001. Scale bars = 200 µm.

To mimic 3D in vivo environments, motor neuron spheroids were cultured to maturity (14 days), resuspended within 3D type I collagen matrices in a concentration dependent manner (Fig. 3a), and again cultured for a further 5 days. Spheroids were then analysed for mRNA markers of maturity (SMI32), vesicular transporter of acetylcholine (VACHT) and microtubule-associated protein tau (MAPT); indicative of axon extension, compared with 14 day monolayer spheroids (Fig. 2). Suspension within 3D collagen environments further enhanced SMI32 mRNA expression, with transcription of this mature gene elevated sixfold in 1 mg/mL hydrogel compositions compared to monolayer (P ≤ 0.001, Fig. 3b). This trend was also evident for the VACHT mRNA, with addition to the 3D environment increasing expression, specifically in 1 mg/mL type I collagen matrices compared to monolayer spheroids (Fig. 3c, P ≤ 0.05). Although not statistically significant, 50% increases in MAPT mRNA were also observed in 1 mg/mL conditions compared to monolayer (Fig. 3d). Consequently, 1 mg/mL concentrations of type I collagen were selected as the ideal loading matrix for iPSC motor neuron spheroids in this work.

### Functional 3D bioengineered primary human skeletal muscle enhances acetylcholine receptor clustering and expression of post-synaptic mRNAs

**Figure 4:**
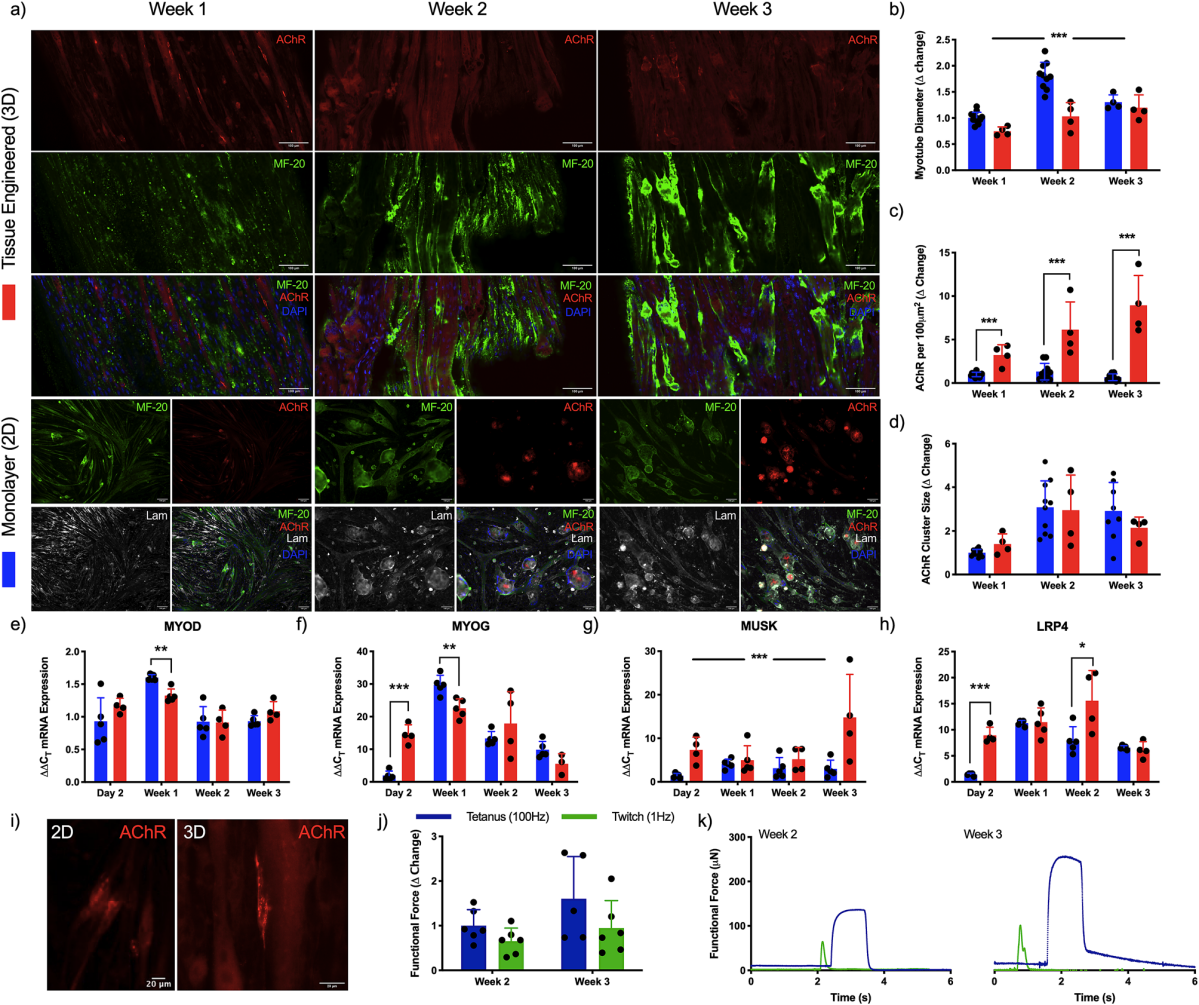
3D culture of primary human skeletal muscle enhances acetylcholine receptor clustering, produces functional force and upregulates transcription of post-synaptic mRNAs. (**a**) Confocal tile scans of single z-plane (Tissue engineered 3D) and fluorescence images (Monolayer 2D) of human skeletal muscle labelled for AChR on the post-synaptic membrane, pan-myosin heavy chain (MF-20), laminin (monolayer only) and nuclear DNA (DAPI) across 1, 2 and 3 weeks culture. Morphological analysis of (**b**) myotube diameter, (**c**) AChR density and (**d**) cluster size. Transcriptional analysis of myogenic; (**e**) MYOD and (**f**) MYOG, and post-synaptic; (**g**) MUSK and (**h**) LRP4 mRNAs. Blue bars = Monolayer (2D), Red bars = Spheroid (3D). (**i**) Zoom images of AChR in monolayer (2D) and tissue engineered (3D) skeletal muscle. (**j**) Functional tetanus and twitch force data and (**k**) representative traces in engineered tissues. Δ change indicative of normalised data to the earliest monolayer time-point. Beginning of representative tetanus and twitch contractions represents initiation of electrical field stimulation, time elapsed before this point is un-stimulated baseline recording. Data presented ± standard deviation (SD). *P ≤ 0.05, **P ≤ 0.01 and ***P ≤ 0.001. Scale bars = (a) 100 µm, (i) 20 µm.

To determine the myogenic development, differentiation of human derived muscle cells (HDMCs) was performed in both monolayer and 3D cultures across a 3 week period to ascertain morphological (Fig. 4a) and functional (Fig. 4j,k) capacity. Further, analysis of myogenic and synaptic mRNA expression, and post-synaptic receptor development were also performed to indicate the optimal window for introduction of the motor nerve input within neuromuscular co-cultures. Differences in myotube diameter were observed between monolayer and 3D tissue, and across the 3 week culture period (P ≤ 0.001). Monolayer cell cultures increased in diameter until week 2 before then reducing in width at week 3, opposed to 3D tissues that demonstrated gradual increases throughout development (Fig. 4b). Linear increases in the number of AChR clusters were evident in both culture modalities, however, these were significantly elevated in 3D tissues at all time-points (P ≤ 0.001, Fig. 4c). Although this increase was not reflected in the size of AChRs (P > 0.05, Fig. 4c), those evident in 3D tissues formed clusters with greater lacunarity as seen in vivo (Fig. 4i)^12^. Transcriptional profiles of MYOD mRNA were consistent across both monolayer and 3D tissues, although elevations in expression were observed after 7 days in monolayer conditions (P ≤ 0.01, Fig. 4e). Expression of MYOG mRNA was significantly elevated after 2 days culture in 3D tissues (P ≤ 0.001), however this decreased compared to monolayer after 1 week in culture (P ≤ 0.01, Fi.g4f.). Increases in MUSK mRNA in 3D tissues was apparent compared to monolayer across the entire culture period (Fig. 4g), whereas LRP4 mRNA was significantly elevated immediately after 2 days (P ≤ 0.001) and again after 14 days culture in 3D tissues (P ≤ 0.05, Fig. 4h). Functional readouts of 3D tissues in this work in response to electrical field stimulation became evident after 2 weeks, with slight increases in force output evident following a further week in culture, however, these changes were not statistically significant (P > 0.05, Fig. 4j,k).

### Loading density of iPSC motor neuron spheroids determines functionality of neuromuscular tissues

**Figure 5:**
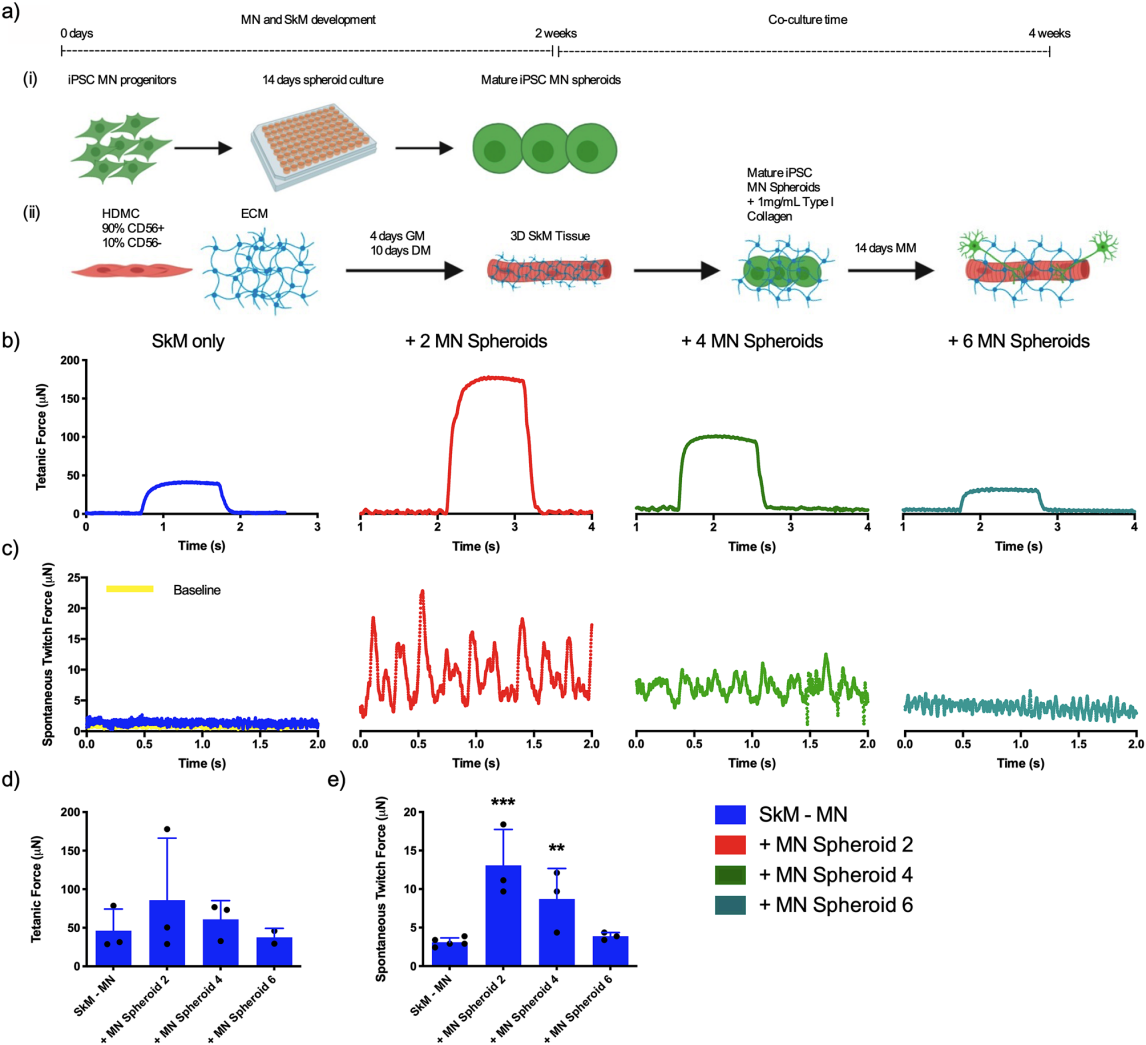
Number of iPSC motor neuron spheroids loaded in tissue engineered skeletal muscle determines functionality of neuromuscular tissues. (**a**) Schematic detailing method of iPSC motor neuron loading to generate neuromuscular tissues at different stages of myogenic development. (i) MN progenitors are loaded to spheroid microplates and matured for 14 days. (ii) Primary HDMCs are seeded in established type I collagen matrix, loaded into 3D printed mould and cultured to maturity for 2 weeks. Matured MNs are then added within optimised type I collagen hydrogel, seeded around SkM and cultured for a further 2 weeks. MN; Motor neuron, SkM; Skeletal muscle, HDMC; Human derived muscle cells, GM; Growth medium, DM; Differentiation medium, ECM; Extracellular matrix, MM; Motor neuron maintenance medium. (**b**) Addition of iPSC motor neurons with type I collagen perimysium at loading densities of 2, 4 or 6 spheroids per tissue evidence tetanic force and (**c**) concentration dependent spontaneous twitch profiles compared to skeletal muscle only tissues. (**d**) Quantification of functional tetanic and (**e**) spontaneous twitch force output with 2, 4 or 6 motor neuron spheroids. Beginning of representative tetanus and twitch contractions represents initiation of electrical field stimulation, time elapsed before this point is un-stimulated baseline recording. Individual functional data points indicative of n = 3 contraction profiles, totalling minimum of n = 9 and maximum of n = 15 per condition and presented ± standard deviation (SD). **P ≤ 0.01 and ***P ≤ 0.001.

Motor neuron spheroids were matured for 14 days as outlined (Fig. 5a,i) and loaded around skeletal muscle tissues at a loading density of 2, 4 or 6 spheroids/tissue after 14 days maturation (Fig. 5a,ii). Following further 2 week co-culture periods, assessment of skeletal muscle only and neuromuscular tissue force production via electrical field stimulation outlined no statistically significant increases in tetanic force when motor neurons were added following 2 weeks of myogenic differentiation (Fig. 5b,d, P > 0.05). Trends in tetanus force were, however, aligned with spontaneously occurring contractions indicative of motor nerve input that were significantly elevated when 2 (P ≤ 0.001) and 4 MN spheroids (P ≤ 0.01) were loaded in surrounding type I collagen perimysium compared to muscle only controls (Fig. 5c,e).

### Addition of iPSC derived motor neuron spheroids to matured bioengineered skeletal muscle produces functional human neuromuscular junctions

**Figure 6:**
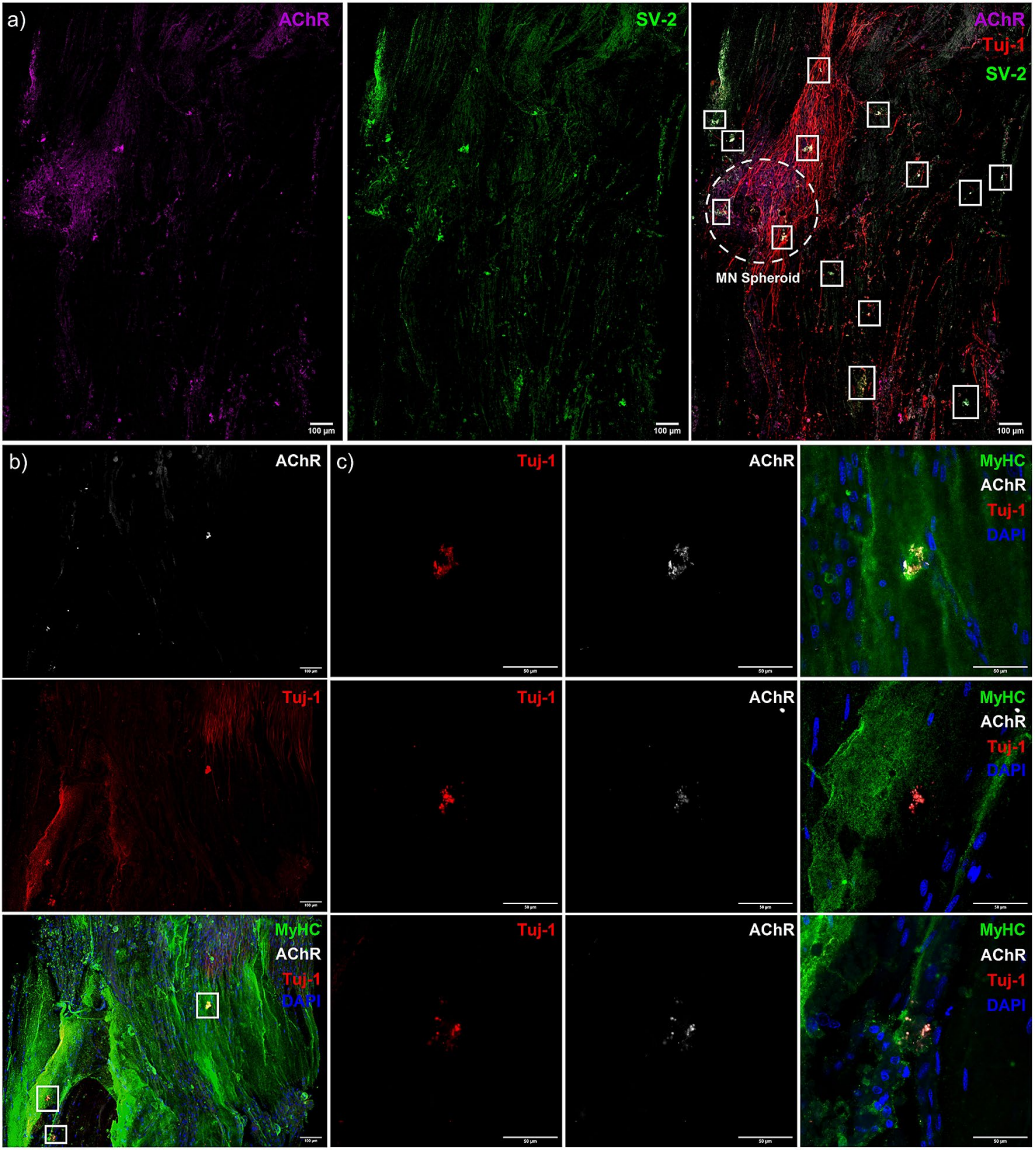
Co-localisation of pre- and post-synaptic membrane proteins, motor nerve axons and myosin heavy chain positive fibres indicate neuromuscular junction formation in engineered tissues. (**a**) Confocal tile scan of neuromuscular tissue detailing AChR (left) and SV-2 (middle), with overlay (right) evidencing multiple synaptic contacts (outlined—white boxes) via co-localisation of pre- and post-synaptic markers and Tuj-1 + neuronal axons. (**b**) Confocal tile scan of neuromuscular tissues evidencing AChR, Tuj-1 + nerve terminal and myosin heavy chain co-localisation. (**c**) Zoom of synaptic contacts highlighted in (**b**) via white box outline. Images were collected across n = 8 neuromuscular tissues. Scale bars = (**a**, **b**) 100 µm, (**c**) 50 µm.

**Figure 7:**
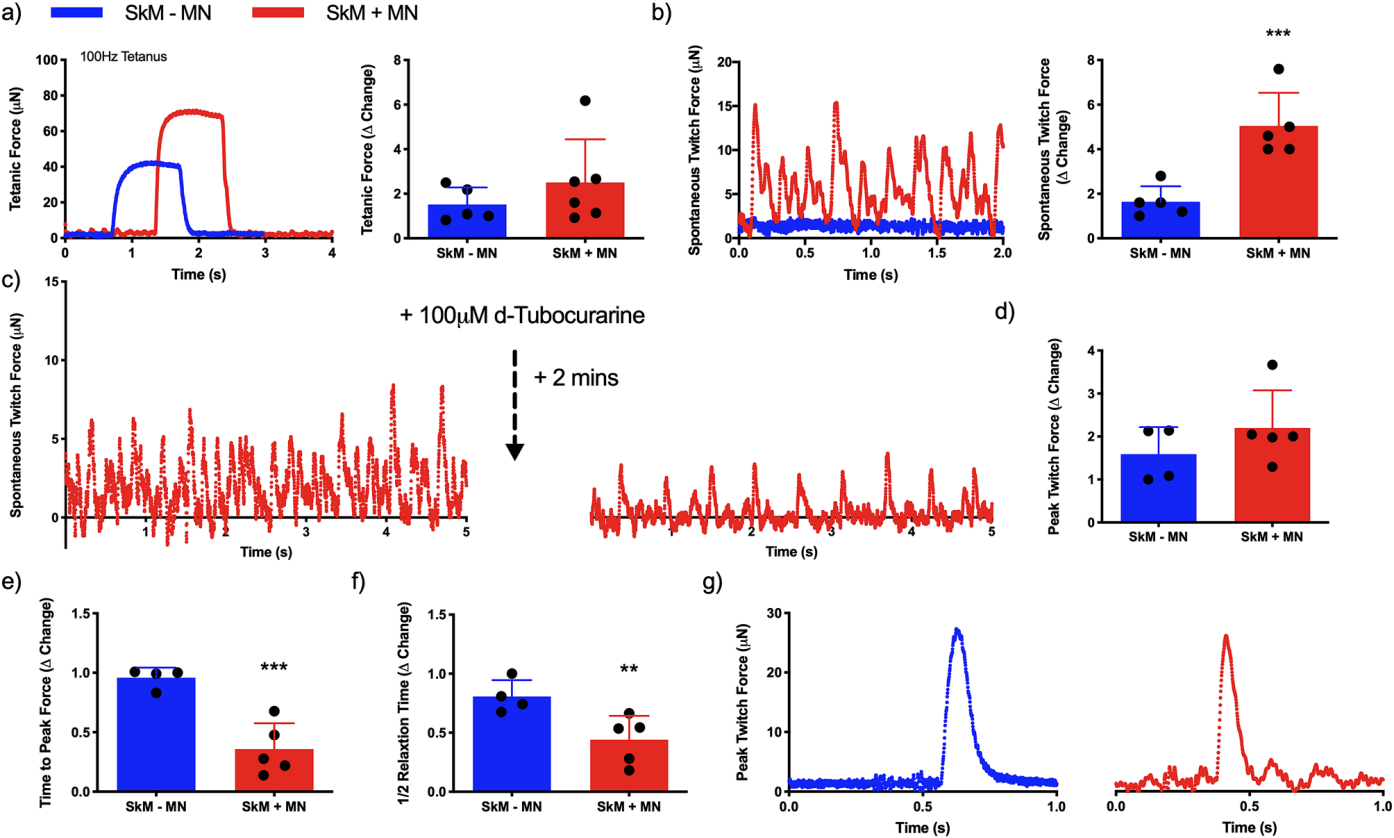
Physiological functionality of engineered tissues is enhanced via neuromuscular junction formation. (**a**) Representative maximal induced tetanus contraction profiles of muscle (SkM -MN) and neuromuscular tissue (SkM + MN) and quantification of tetanic force. (**b**) Spontaneously occurring twitch force profiles in engineered tissues with (SkM + MN) and without (SkM–MN) iPSC motor neuron spheroids, and quantification of spontaneous twitch force driven via motor nerve inclusion. (**c**) Inhibition of the acetylcholine receptor in spontaneously contracting neuromuscular tissues via addition of 100 µM d-Tubocurarine. (**d**) Peak twitch force induced via electrical field stimulation. (**e**) Time to peak twitch force and (**f**) half relaxation time as physiological measures of contractile function. (**g**) Representative twitch profiles evidencing reduced time to peak twitch and shorter relaxation profiles in SkM + MN tissues. Δ change indicative of normalised data to SkM–MN. Beginning of representative tetanus and twitch contractions represents initiation of electrical field stimulation, time elapsed before this point is un-stimulated baseline recording. Individual functional data points indicative of n = 3 contraction profiles per tissue, totalling minimum of n = 12 and maximum of n = 18 contractions per condition and presented ± standard deviation (SD). **P ≤ 0.01 and ***P ≤ 0.001.

Confocal tile scans of neuromuscular tissues confirmed innervation indicated by co-localisation of the pre- [synaptic vesicle protein 2 (SV-2)] and post-synaptic (AChR) markers, and motor nerve axons (Tuj-1, Fig. 6a). Neuronal axons tracking along myofibers positive for myosin heavy chain and colocalising with AChR clusters (Fig.S6b) were also evident, in addition to presenting classical pretzel-like morphologies (Fig. 6b,c). Functionality of skeletal muscle tissue was then assessed via electrical field stimulation (tetanus and peak twitch), while functional synaptic contacts were confirmed via analysis of spontaneously occurring twitch profiles (no electrical field stimulation) and antagonistic blocking of the AChRs. No significant differences were observed in tetanic force despite addition of the motor neurons (P > 0.05, Fig. 7a), however, significant increases in spontaneous twitch force were evident (P ≤ 0.001, Fig. 7b). Muscular tissue devoid of the motor nerve displayed spontaneous contractions comparable to baseline readings, which was increased fourfold above baseline in neuromuscular tissues. To fully determine functionality between pre- and post-synaptic membranes, the AChR antagonist d-tubocurarine was added to spontaneously contracting tissues (Fig. 7c). Following addition, total twitches and frequency of twitch profiles were ablated indicating inhibition of the AChR and confirming motor nerve induced contractions in neuromuscular tissues. Comparable peak twitch profiles were also evident in both skeletal muscle only and neuromuscular tissues (P > 0.05, Fig. 7d). However, measures of skeletal muscle excitability; time to peak twitch (P ≤ 0.001, Fig. 7e), and relaxation; half relaxation time (P ≤ 0.01, Fig. 7f), were significantly decreased following innervation (Fig. 7g).

## Discussion

New technologies are required that enable the study of the human NMJ in development, throughout disease progression and in response to therapeutic interventions. This work responds to this need, utilising a freely available 3D printed system to bioengineer human skeletal muscle and provides the methodologies to incorporate commercially available iPSC derived motor neuron progenitors to create functional human NMJs^34^.

iPSC motor neuron progenitors were cultured as 3D spheroids to enhance maturity and contain cellular soma separate from skeletal muscle tissues. Typical spheroid cultures utilise cell–cell adhesion to form multicellular aggregates that secrete extracellular matrix proteins^35^. Enhanced transcription of motor neuron lineage markers in neural stem cell spheroids compared to monolayer cells has previously been reported^36^. Our data supports this methodology, evidencing enhanced expression of developmental, progenitor, motor neuron and mature mRNAs, in addition to decreased proliferation of motor neuron progenitor spheroids compared to monolayer (Fig. 1). Immunostaining for progenitor and motor neuron markers Olig-2 and HB-9, showed preferential localisation of these proteins to the perimeter of motor neuron spheroids. HB-9 localisation to spheroid perimeters has been previously observed, with a normoxic-necrotic gradient evident from perimeter to core^28^. This potentially indicates a less preferential cellular environment in the core of motor neuron spheroids used in this work. However, the augmented transcription of OLIG2, ISLET1 and SMI32 mRNAs, ~ 400, ~ 150 and ~ 200-fold respectively, observed in spheroid cultures indicates that the neurodifferentiation achieved is significantly advanced when compared to monolayer equivalents, regardless of these potential normoxic-necrotic gradients. Axon projections of cultured spheroids after 7, 14 and 21 days demonstrated preference for specific developmental stages (Fig. 2). Maximal axon extension was observed after 14 days spheroid culture, aligning with mRNA expression data. Transcription of OLIG2 and ISLET1 increased linearly until day 14 before reducing significantly by day 21, with only mature marker SMI32 further enhancing transcription at this time-point. This suggests that spheroids matured in suspension, as used in this work, require adherent surfaces prior to this point for optimal neurite growth. Differences in axon projections of neuronal stem cell and progenitor spheroids have been observed previously, with maximal axon lengths of < 400 µm being enhanced to ~ 800 µm following vascular integration^36^. Axon lengths of ~ 700 µm have also been evidenced in spheroids devoid of vasculature in 3D microfluidic devices^28^. Importantly, this methodology to produce 3D spheroids results in axons, at 14 days, of 1000–1600 µm length of which 90% are SMI-32+ (Fig. 2) in the absence of vasculature.

In vivo the spinal motor neuron soma resides within a 3D environment of grey matter within the ventral horn, extending axons to the peripheral musculature. In an attempt to replicate this environment, different concentrations of type I collagen hydrogels were utilised to manipulate matrix properties. Increases in the transcription of genes indicative of maturity (SMI32), axon extension (MAPT) and vesicular transport of ACh (VACHT) neurotransmitter were evident in spinal motor neuron spheroids across type I collagen concentrations. This outlines that maturity and neurotransmitter function can be enhanced via addition to optimised 3D environments, and that the matrix composition, which differs from that required for skeletal muscle, affects expression of key mRNAs (Fig. 3). Enhanced differentiation and axon growth of iPSC motor neuron spheroids has also been reported in soft methacrylated hyaluronic acid hydrogel compositions^37^. However, previous work to load motor neuron spheroids to muscle tissue has used 2.4 mg/mL concentrations of type I collagen and 2 mg/mL supplemented with Matrigel in 4:1 ratios^28,29^. This is contrasting to our data that demonstrates enhanced biological transcription at 1 mg/mL, decreasing toward that of monolayer spheroids at lower (0.5 mg/mL) and higher (1.7 mg/mL) concentrations. This matrix concentration provides an optimised hydrogel for 3D culture of iPSC motor neuron spheroids and as a delivery vehicle to engineered muscle tissues using polymerised type I collagen. Further examples of tailored 3D matrices for iPSC lower motor neurons are currently limited. However, the preferential responses observed at 1 mg/mL concentrations in this work indicates that this area requires additional methodological consideration when creating 3D neurological or neuromuscular systems. Here, refining this matrix delivery system further via inclusion of influential neural extracellular proteins may promote enhancements in maturity and biological functionality for 3D motor neuron spheroid cultures^38^.

Multiple methods to bioengineer human skeletal muscle now exist using both primary and stem cell myogenic precursors^34,39–41^. Consistent with previous research, 3D skeletal muscles in this work demonstrated enhanced numbers of AChR clustering across 3 week culture periods and representative pretzel-like morphologies compared to monolayer (Fig. 4)^30^. This acceleration in development is mirrored in synaptic mRNAs for MuSK and Lrp4, which associate at the NMJ with z-agrin to form a protein complex across the synaptic basal lamina^1,2^. Significant increases in MUSK mRNA were evident compared to monolayer throughout myogenic development, specifically notable after 2 days and 3 weeks culture. This may underpin the increased AChR clustering and morphological maturation observed in 3D tissues, as MuSK is known to regulate AChR clustering and organisation of the post-synaptic membrane in mice^42,43^. This initial increase in transcription is also apparent in LRP4 mRNA, however, comparable levels are evident in monolayer myotubes after 1 week. At this point, monolayer expression has peaked, while mRNA levels in 3D tissue rise significantly until 2 weeks culture before also decreasing. Together, this data indicates upregulation of mRNA transcription for translation of the ‘core’ proteins expressed by the muscle required for NMJ formation and maintenance, and development of the post-synaptic apparatus for motor nerve synaptogenesis in 3D tissues after 2 weeks myogenic development.

Neuromuscular development in skeletal muscle is a multifaceted process of primary and secondary motor innervation that is highly dependent on myogenic status^3^. Myogenic progenitors in the paraxial mesoderm either side of the neural tube proliferate and fuse to form immature primary myotubes^44^. Primary myotube developmental stages precede that of axon penetration from the cervical and brachial plexus in the diaphragm of mice and rats^45,46^. Following primary innervation, residual fetal myoblasts fuse and generate secondary myotubes^44^. However, the formation of secondary fibres requires the presence of the motor nerve, which influences post-synaptic specialisation such as contractile properties of fibres^47^. Previous work has introduced the motor nerve at experimental onset prior to primary myotube formation and following initial myogenesis, both generating functional neuromuscular tissues^28,30^. In our model, attempts to incorporate motor neuron spheroids with HDMCs at experimental onset did not form functional tissues. However, this is likely due to the requirement for complex medium formulations that simultaneously support differentiation phases of both cell types, opposed to developmental issues (Fig. S4). Engineered tissues in this work fuse to form functional primary myotubes after 14 days culture in the absence of motor axons. At this point, motor neuron addition enables innervation of myotubes within a further 2 week culture period. The paucity of data regarding human NMJ formation provides difficulties when devising methodologies that accurately represent neuromuscular development in vitro*.* However, systems that enable the precise addition of motor neurons dependent on myogenic status, as reported in this work, likely afford the greatest utility in this endeavour.

Motor recruitment of skeletal muscle fibres in vivo is performed in a size dependent manner by α motor neurons^48^. Typically, singular motor neurons regulate recruitment of multiple groups of fibres via axonal branching comprising one motor unit, with multiple motor units necessary to maximally contract entire muscles. To investigate the motor pool required to maximally contract engineered muscle tissues in this work, motor neuron spheroids were loaded, following 2 weeks maturation (muscle and motor nerve), at ratios of 2, 4 or 6 per tissue. Negative linear relationships in spontaneous muscle contractions were observed indicating a maximal capacity for spheroid addition in this model (Fig. 5). Regarding previous human NMJ models, monolayer cell clusters were loaded to engineered muscle tissues at a ratio of 3 per ~ 10 mm tissue^30^. Our data aligns with these ratios, indicating maximal fibre recruitment when loading 1 motor nerve per ~ 3 mm of engineered muscle tissue length. Importantly, this data also evidenced a threshold of 1 spheroid per ~ 1 mm of muscle tissue length at which innervation and or motor recruitment appears inhibited. This is contrasting to that observed in the microfluidic platform to create neuromuscular tissues, that loaded a singular motor spheroid per muscle tissue of approximately 1.5 mm lengths^28^. . This may allude to competition for nutrient and or growth factor availabilities as the major determinant. Motor neuron spheroids are matured individually in 200 µL volumes, with motor unit tissues residing in 2 mL of supplemented medium. This would provide adequate growth factor concentrations even at highest spheroid ratios. However, given the introduction of the muscle tissue that has also been known to utilise neuronal growth factors such as BDNF in mice, further research is required to establish the biochemical or physical causality of this effect^49^.

High levels of pre- and post-synaptic co-localisation with adjacent neuronal axons were apparent in neuromuscular tissues with ~ 13 synaptic contacts evident within a singular z-slice, equating to 4.6 NMJs per mm^2^. High magnification images of these contacts showed features consistent with human NMJ morphologies, in addition to Tuj-1 + nerve axons tracking along myosin heavy chain positive fibres (Fig. 6). Although Tuj-1 is a pan neuronal marker, given our earlier developmental and motor neuron characterisation data (> 90% axons SMI-32 + Day 14) we are confident that the co-localisation of Tuj-1 with markers of pre- (SV-2) and post-synaptic (AChR) functionality indicates formation of neuromuscular synapses in our model. Human NMJ models must enable such morphological and functional characterisation to provide complete utility in physiological and or pathophysiological studies, in addition to sensitive protein and RNA analyses. Previous studies have provided functional data, yet limited morphological characterisation other than adjacent Tuj-1 positive axons and muscle fibres^28,29^. The most sophisticated morphological characterisation of 3D tissues to date have identified core synaptic proteins of laminin-β2, MuSK and rapsyn at the motor nerve-muscle interface^30^. Although thorough functional analysis was also performed, all aforementioned proteins are either expressed or accumulated by the post-synaptic membrane^3^. The vast majority of immunohistology from in vivo (human, mice) samples use a combination of 2H3/SV-2 for neuronal pre-synaptic identification with AChR co-localisation^14^, and consequently to ensure consistency of biochemical characterisation within 3D tissue engineered systems it is recommended to also label proteins of both pre- and post-synaptic functionality (AChR and synaptic vesicle markers; synaptotagmin, SV-2).

Physiological assessments of motor unit function confirmed spontaneous contraction profiles were driven via the NMJ with inhibition of the post-synaptic membrane using AChR antagonists. This method has widely been utilised amongst the literature in vitro^22,26^. Notably, no significant differences in induced maximal tetanic or peak twitch contraction forces were evident in innervated tissues. This aligns with microfluidic human 3D neuromuscular co-cultures, but contrasts with those produced using primary rat derived myogenic and motor neuron cells^26,28^. This work does, however, evidence that NMJ formation within bioengineered human motor units enhances the neuromuscular physiology measures; time to peak twitch and ½ relaxation time, typically used to assess neuromuscular function in vivo^50^. Following the release of ACh and subsequent binding to the AChR, the resulting depolarisation opens voltage gated sodium ion channels creating an action potential that propagates along the cell, invades the t-tubules and elicits opening of L-type calcium channels. Ryanodine receptors (RyRs) in the sarcoplasmic reticulum (SR) then open and release calcium required for muscular contraction. Reductions in cytosolic calcium is then achieved via SR re-uptake mediated the sarco- endoplasmic reticulum calcium ATPase (SERCA) pump eliciting muscle relaxation^4^. Time to peak twitch is typically indicative of skeletal muscle excitability, which is underpinned by the efficiency of neuromuscular transmission, propagation of action potential and release of calcium via the SR RyRs. Whereas decreased half relaxation time has been outlined to be indicative of calcium re-uptake in the SR via the SERCA pump, with increased SERCA1 mRNA transcription evident following electrical stimulation of 3D muscle tissue^51^. Both functional measures were significantly decreased in time following innervation, indicating an enhanced efficiency of the molecular mechanisms of muscle contraction. Importantly, neuromuscular tissues using primary rat cells reported increases in time to peak force and half relaxation time measures upon innervation, suggesting that alterations in the excitability of bioengineered motor units appears sensitive across species. This further outlines the importance of models derived entirely from human cellular material for the study of cell/molecular biology and physiology of the human neuromuscular junction.

## Conclusion

In summary, this work presents the methods required to maximise the maturity of both iPSC motor neurons and primary skeletal muscle, utilising cell type specific extracellular matrices and developmental timelines to bioengineer the human motor unit for the study of neuromuscular physiology. Future work should seek to compare this model against in vivo NMJ samples obtained from experimental rodents and humans, to confirm the utility of this system to accurately represent the intricate differences in human NMJ physiology compared to experimental animals. Focus should also be applied to the integration of additional neuronal cell types such as non-myelinating and myelinating schwann cells, and sensory neurons to complete the neuromuscular reflex-arc. Finally, this model should be translated to provide a functional assay of NMJ pathophysiology via integration of patient iPSC motor neurons.

## Materials and methods

### Primary human derived myoblast cell purification and culture

This study was approved by the Loughborough University Ethics Approvals (Human Participants) Sub Committee (reference number: R18-P098) and all experiments were performed in accordance with these guidelines/regulations. Healthy male subjects provided written informed consent and completed a medical screening questionnaire prior to participation. Primary HDMC samples were obtained from healthy males (n = 3), between the ages of 18–55 reporting no recent injuries or intake of anti-inflammatory pharmaceuticals, from the vastus lateralis muscle using routine muscle biopsy procedure with micro-biopsy. HDMCs were isolated using an established explant protocol and sorted for the expression of CD56, with detailed methods of the biopsy procedure and cell purification methodology being available in the Supplementary Information. Upon resuscitation, HDMC CD56 + and − cells were expanded separately until passage 7/8 for use then remixed at a ratio of 9:1 (+ /−). Remixed HDMCs were plated for experimental use in monolayer at 1 × 10^4^ cells/cm^2^ and cultured in growth medium (GM); composed of 79% Dulbecco’s modified Eagles medium (DMEM; Sigma), 20% fetal bovine serum (FBS, Pan Biotech) and 1% penicillin–streptomycin (P/S, Fisher) until confluence, prior to inducing differentiation via addition of differentiation medium (DM) composed of; 97% DMEM, 2% horse serum (Sigma), 1% P/S supplemented with 20 ng/mL insulin-like growth factor 1 (IGF-1, PeproTech). HDMC medium was replenished entirely at 48 h intervals.

### iPSC derived motor neuron monolayer and spheroid culture

Following resuscitation, iPSC derived motor neuron progenitors (Axol Bioscience, UK, ax0078) were seeded at 2 × 10^6^ cells/T25 culture flask coated with 1.5 mg/mL Corning Matrigel (Fisher) DMEM solution, in motor neuron recovery medium (RM, Axol) supplemented with 0.1 µM all-trans retinoic acid (RA, Sigma) and 10 µM Y-27332 ROCK inhibitor (Stem Cell Technologies, removed after 24 h). At cellular confluence, motor neuron progenitors were dissociated using TrypLE select (1x, Gibco, Fisher), centrifuged at 200 G for 5 min and seeded in 96-well U bottom spheroid microplates (MoBiTec, MS-9096UZ) at 1 × 10^4^ cells/well or monolayer at 1 × 10^5^ cells/cm^2^ in motor neuron maintenance medium (MM, Axol) supplemented with 0.5 µM RA, 5 ng/mL brain derived neurotrophic factor (BDNF, PeproTech) and 10 ng/mL ciliary neurotrophic factor (CNTF, Axol). Monolayer motor neuron progenitors MM for cell seeding was supplemented with 10 µM Y-27332 ROCK inhibitor to promote adhesion and survival. All medium for both monolayer and 3D spheroids were changed completely after 24 h and then replenished by 50% (half-change) thereafter at 48 h intervals. Monolayer and spheroid motor neurons were isolated for mRNA analyses at 2, 7, 14 and 21 days of maturation. For analysis of motor neuron spheroid axonal extension at varying developmental stages (7, 14 and 21 days), spheroids were removed from microplate wells, individually seeded in complete MM on Matrigel coated (1.5 mg/mL) well plates. Motor neuron spheroids were observed to fully extend axons after 72 h in culture, and were then cultured for a further 48 h to ensure a plateau in axon length was achieved, totalling 5 days adherent culture prior to fixation.

### 3D skeletal muscle and motor neuron tissue engineering

Skeletal muscle tissue engineering was undertaken as previously reported via addition of 65% v/v type I rat tail collagen; dissolved in 0.1 M acetic acid, protein at 2.035 mg/mL (First Link, UK), with 20% Matrigel matrix and 10% v/v of 10 × MEM (Fisher)^34^. This solution was neutralized by the dropwise addition of 5 and 1 M sodium hydroxide (NaOH, Sigma), until a colour change to cirrus pink was observed. HDMC cells were added at a seeding density of 4 × 10^6^ cells/mL in a 5% v/v GM solution to 3D printed moulds and incubated for 10–15 min (37 °C, 5% CO_2_). Engineered skeletal muscles were then maintained in 2 mL GM and cultured for a further 14 days (4 days GM, 10 days DM). To optimise the preferred neuronal type I collagen matrix concentration, iPSC motor neuron spheroids were removed from microplates and added to a pre-neutralised stock collagen/MEM hydrogel solution (1.73 mg/mL) further diluted with MM to yield the following concentrations: 1.7 mg/mL, 1 mg/mL and 0.5 mg/mL at 500µL volumes in culture well plates. Following neutralisation, each 3D motor neuron hydrogel was maintained in MM and cultured alongside monolayer spheroid conditions, prepared as previously described, and cultured for 5 days prior to isolation for genetic and morphological analyses. Spheroids were also cultured in monolayer on 1.7 mg/mL, 1 mg/mL and 0.5 mg/mL coated surfaces, however this reduced adherence and axonal extension compared to Matrigel coated plates (Fig. S3).

### 3D neuromuscular tissue engineering

Engineered primary HDMC tissues are cultured for 14 days as described above, prior to being encased within an additional extracellular matrix, comparable in hierarchy to the perimysium, that contains iPSC derived motor neuron spheroids. The maturity of the motor neuron spheroids is standardised at the optimised time-point (14 days) for axonal extension and maturation as is the co-culture time-period (14 days). To combine motor neuron spheroids into appropriate hydrogel volumes/concentrations, spheroids are extracted and placed at desired numbers in sterile Eppendorf tubes, centrifuged at 100 G for 30 s, with the supernatant discarded prior to resuspension within desired loading matrix (Fig.S1). HDMC tissues reside within 3D printed rectangular moulds that are adhered to standard cell culture well-plates. All medium in the culture well and any remaining within the rectangular mould is removed. To create the perimysium structure and load motor neuron spheroids, the optimised type I collagen (1 mg/mL) matrix containing motor neuron spheroids is then pipetted into the rectangular mould, encasing pre-differentiated HDMC tissues (day 14) that are then cultured for a further 14 days within MM (Fig. S2) to form motor units.

### Functional assessment of skeletal muscle and neuromuscular tissues

Engineered skeletal muscle and neuromuscular tissues were washed twice in phosphate buffered saline (PBS) prior to one end of the engineered muscle being lifted from the pseudo tendon pin. The loose end of the construct was then attached to the force transducer (403A Aurora force transducer, Aurora Scientific) using the eyelet present in the construct. For direct electrical stimulation, the tissue was submerged (3 mL) in Krebs–Ringer-HEPES buffer solution (KRH; 10 mM HEPES, 138 mM NaCl, 4.7 mM KCl, 1.25 mM CaCl_2_, 1.25 mM MgSO_4_, 5 mM Glucose, 0.05% Bovine Serum Albumin in dH_2_O). Wire electrodes were positioned parallel either side of the construct to allow for electric field stimulation. Impulses were generated using LabVIEWsoftware (National Instruments, UK) connected to a custom-built amplifier. Peak twitch force was determined using a single 3.6 V/mm, 1.2 ms impulse and maximal tetanic force was measured using a 1 s pulse train at 100 Hz and 3.6 V/mm, generated using LabVIEW 2012 software (National Instruments, UK). For measurement of spontaneous muscle contraction driven by the motor nerve in neuromuscular tissues, once attached, each tissue sample was recorded for a period of ≥ 30 s with no electrical stimulation. Antagonistic blocking of the AChR was achieved via 1 mL bolus of 200 µM d-tubocurarine (Sigma) to 1 mL of surrounding basal medium (MM), achieving 100 µM concentration as previously reported^26^. Electrical field stimulation to obtain peak twitch and tetanus data were derived from 3 contractions per tissue sample with standardised separation times of 10 s between contractions. For each representative trace (tetanus and twitch), the beginning of contraction represents the initiation of electrical field stimulation, with time elapsed before this point displaying un-stimulated baseline recording*.* Any visual difference in time before the beginning of tetanus or peak twitch traces is an artifact of graphical representation when extracting the data and has no experimental or scientific implications*.* All data was acquired and analysed using a Powerlab system (ver. 8/35) and associated software (Labchart 8, AD Instruments, UK).

### Immunocytochemistry and morphological analysis

To perform immunocytochemistry at experimental termination, monolayer and 3D samples were washed twice in 2 mL of PBS/well and fixed using a 3.7% paraformaldehyde solution (Sigma). Monolayer and 3D samples were then permeabilised (0.2% Triton X-100, Fisher) and blocked using 5% goat serum (Fisher) for 30 min, prior to being incubated overnight (≥ 12 h) with primary antibody solutions. Primary antibodies were co-incubated with 5% goat serum to reduce non-specific binding. Labelled samples were then counterstained with secondary antibody fluorophores and fluorescent small molecules for ≥ 2 h (Table S1). Samples were washed 3 times (1 × tris-buffered saline (TBS)) before permeabilisation and after both primary and secondary antibody incubations.

Fluorescence images were captured using a Leica DM2500 fluorescence microscope with manufacturer’s software (Leica Application Suite X). Confocal imaging was undertaken on a Zeiss LSM 880, using 40 × and 63 × oil objectives. Images captured via confocal tile scan are stitched together enabling visualization of large tissue sections. One standard scan equates to a 3 × 7 tile image, taken in a single z-plane from the centre of the engineered tissue. Scans used to identify synaptic contacts (AChR:SV-2:Tuj-1 and AChR:Tuj-1:MF-20) and co-localisation in neuromuscular tissues are 10 × 8 and 7 × 5 stitched visualisations respectively. Images were analysed using IMAGE J 1.50a/Fiji (Java 1.6.0_24) software (National institute of Health, USA)^52^. Monolayer image inclusion criteria (n = 1) were set at ≥ 5 images taken at random locations per well. Myotube inclusion criteria were defined as containing ≥ 3 nuclei per myotube; 2 nuclei constituting a dividing cell and 3 or more indicating myogenic fusion. Myotube width analysis was performed via measuring the central and representative region of each myotube within an image. Analysis of AChR size and number was performed using an in-house macro designed for Fiji (Java 1.6.0_24) image analysis software (Image J 1.50a). Briefly, the macro converts images to 8-bit, applies a standardised threshold and then utilises the analyse particles plugin to create a mask. From this mask the data is automatically obtained based on the set measurements within the macro. AChR number is obtained by the amount of receptor clusters that are analysed, with the size determined by the area of each cluster analysed. Analysis of motor neuron axonal extension in spheroids at 7, 14, and 21 days maturation was undertaken using a coordinate analysis system. Coordinates are assigned to the centre of the spheroid and to all adjacent axon terminations. Axon termination X and Y coordinates are then subtracted from respective central equivalents to yield axon length (µm). To determine change in axon length over time, all data is normalised to the earliest developmental time-point (7 + 5 days). This coordinate system is then applied at each time point to both Tuj-1 + and SMI-32 + axons respectively, to ascertain axon lengths immunolabelled for each marker. SMI-32 + axon lengths are then expressed as a percentage of Tuj-1 + axon projections to provide an indication of axon maturity extending from the spheroid.

### RNA extraction and quantitative real-time polymerase chain reaction

Tissue-engineered skeletal muscle was thawed and suspended in 500μL TRIzol (Fisher) before being homogenized using TissueLyserII (Qiagen) for ≥ 2 min until tissue degradation was complete. Monolayer and motor neuron spheroid RNA was extracted using the TRIzol method, according to manufacturer’s instructions (Sigma). RNA concentration and purity were obtained by UV–Vis spectroscopy at optical density of 260 and 280 nm using a Nanodrop 2000 (ThermoFisher Scientific). All RNA samples were analysed in duplicate. Five nanograms of RNA were used per real-time polymerase chain reaction (RT-PCR) for RPIIβ, PAX6, OLIG2, ISLET1, SMI32, MKI67, MAPT, VACHT, MYOD, MYOG, LRP4 and MUSK (Table S2). RT-PCR amplifications were carried out using a Power SYBR Green RNA-to-CT 1 step kit (Qiagen) on a ViiATM Real-Time PCR System (Applied Biosystems, Life Technologies), analysed using ViiATM 7RUO software. The RT-PCR procedure was as follows: 50 °C, 10 min (for cDNA synthesis), 95 °C, 5 min (transcriptase inactivation), followed by 95 °C, 10 s (denaturation), 60 °C, 30 s (annealing/extension) for 40 cycles. Relative gene expression was calculated using the comparative C_T_ (ΔΔC_T_) equation for normalized expression ratios; relative expression calculated as 2 − ΔΔC_T_, where C_T_ is representative of the threshold cycle^53^. RPII-β was used as the reference gene in all RT-PCR assays. To compare conditions, one control sample from each experimental repeat (n = 3) was used as the calibrator condition in the C_T_ (ΔΔC_T_) equation. RT- PCR data is presented as the relative gene expression level, determined by the ΔΔC_T_ equation.

### Statistical analyses

Significance of data were determined using IBM SPSS Statistics version 23. Mauchly’s test of sphericity and Shapiro–Wilk tests were used to confirm homogeneity of variance and normal distribution of data respectively. Where parametric assumptions were met, factorial analysis of variance (ANOVA) was performed with Bonferroni post hoc analyses used to analyse differences between conditions at specific time-points. Nonparametric Kruskal–Wallis (H) analysis was undertaken where data violated parametric assumptions. Mann Whitney (U) tests were then utilised to determine the significance between conditions, in accordance with Bonferroni correction to account for incremental type-1 error. All data is reported as mean ± standard deviation (SD). Statistical significance was assumed at P ≤ 0.05.

### Ethics approval and consent to participant

This study was approved by the Loughborough University Ethics Approvals (Human Participants) Sub Committee (reference number: R18-P098). Healthy male subjects provided written informed consent and completed a medical screening questionnaire prior to participation.

## Supplementary Information

Supplementary Information.

## Data Availability

The datasets supporting the conclusions of this article are included within the article and its additional files. Raw data is available from the corresponding author upon reasonable request.
